# Variation of Chromosome Composition in a Full-Sib Population Derived From 2x × 3x Interploidy Cross of *Populus*

**DOI:** 10.3389/fpls.2021.816946

**Published:** 2022-01-26

**Authors:** Yu-Hang Zhong, Yun-Fei Zheng, Yin-Xuan Xue, Lv-Ji Wang, Jin-Wang Zhang, Dai-Li Li, Jun Wang

**Affiliations:** ^1^National Engineering Research Center of Tree Breeding and Ecological Remediation, Beijing Forestry University, Beijing, China; ^2^Key Laboratory of Genetics and Breeding in Forest Trees and Ornamental Plants, MOE, Beijing Forestry University, Beijing, China; ^3^The Tree and Ornamental Plant Breeding and Biotechnology Laboratory, National Forestry and Grassland Administration, Beijing Forestry University, Beijing, China; ^4^College of Biological Sciences and Technology, Beijing Forestry University, Beijing, China; ^5^Forestry and Grassland Research Institute of Tongliao City, Tongliao, China; ^6^Beijing Institute of Landscape Architecture, Beijing, China

**Keywords:** aneuploidy, chromosome composition, heterozygosity transmission, interploidy cross, *Populus*, SSR karyotypic analysis, 45S rDNA–FISH

## Abstract

Interploidy cross commonly results in complex chromosome number and structural variations. In our previous study, a progeny with segregated ploidy levels was produced by an interploidy cross between diploid female parent *Populus tomentosa* × *Populus bolleana* clone TB03 and triploid male parent *Populus alba* × *Populus berolinensis* ‘Yinzhong’. However, the chromosome compositions of aneuploid genotypes in the progeny were still unclear. In the present study, a microsatellite DNA allele counting–peak ratios (MAC-PR) method was employed to analyze allelic configurations of each genotype to clarify their chromosome compositions, while 45S rDNA fluorescence *in situ* hybridization (FISH) analysis was used to reveal the mechanism of chromosome number variation. Based on the MAC-PR analysis of 47 polymorphic simple sequence repeat (SSR) markers distributed across all 19 chromosomes of *Populus*, both chromosomal number and structural variations were detected for the progeny. In the progeny, 26 hypo-triploids, 1 hyper-triploid, 16 hypo-tetraploids, 10 tetraploids, and 5 hyper-tetraploids were found. A total of 13 putative structural variation events (duplications and/or deletions) were detected in 12 genotypes, involved in chromosomes 3, 6, 7, 14, 15, 16, and 18. The 46.2% (six events) structural variation events occurred on chromosome 6, suggesting that there probably is a chromosome breakpoint near the SSR loci of chromosome 6. Based on calculation of the allelic information, the transmission of paternal heterozygosity in the hypo-triploids, hyper-triploid, hypo-tetraploids, tetraploids, and hyper-tetraploids were 0.748, 0.887, 0.830, 0.833, and 0.836, respectively, indicating that the viable pollen gains of the male parent ‘Yinzhong’ were able to transmit high heterozygosity to progeny. Furthermore, 45S rDNA–FISH analysis showed that specific-chromosome segregation feature during meiosis and chromosome appointment in normal and fused daughter nuclei of telophase II of ‘Yinzhong,’ which explained that the formation of aneuploids and tetraploids in the progeny could be attributed to imbalanced meiotic chromosomal segregation and division restitution of ‘Yinzhong,’ The data of chromosomal composition and structural variation of each aneuploid in the full-sib progeny of TB03 × ‘Yinzhong’ lays a foundation for analyzing mechanisms of trait variation relying on chromosome or gene dosages in *Populus*.

## Introduction

Chromosome number variation, such as euploidy and aneuploidy variation, commonly leads to extensive phenotypic changes, which have been widely used in breeding programs of plants. Compared to euploids, aneuploids characterized by loss or gain of one or more individual chromosome(s) are particularly valuable for chromosome engineering breeding and cytogenetic research. According to the loss or gain of chromosome(s) from euploid, aneuploid commonly can be classified into two basic types: hypo- and hyper-euploid. Both types of aneuploids could be produced by crossing with polyploids because imbalanced meiotic chromosome segregation and chromosome elimination of polyploids can result in the formation of aneuploid gametes. In *Arabidopsis thaliana*, Henry et al. (2005) produced a swarm of different aneuploids by self-pollination of triploids, and revealed that chromosome composition and dosage variation strongly result in phenotypic changes (Henry et al., 2010). In apple, aneuploids with 35–55 chromosomes and tetraploids with 68 chromosomes were also produced from the crosses with diploids and triploids (Zhang and Park, 2009). To analyze the chromosomal function and evolutionary relationships and to locate functional genes, serial lines of monosomes, nullisomes, trisomes, and tetrasomes have all been established in wheat (Law et al., 1987). Undoubtedly, chromosome manipulation based on aneuploids has attracted increasing attention for improving the efficiency in plant breeding.

*Populus* is an important model for the forest tree biotechnology and genetic research (Taylor, 2002; Wullschleger et al., 2002; Jansson and Douglas, 2007). The utilization of chromosome number variation, especially polyploidization, has been proved as an important approach in *Populus* breeding programs. In the past several decades, many allotriploid poplar cultivars, such as *P.* × ‘Beilinxiongzhu 1,’ *P. tomentosa* ‘Sanmaoyang,’ *P. tremula* × *P. tremuloides* cv. Astria, *P.* × *euramericana* ‘Zhonglin-46,’ and *P. alba* × *P. berolinensis* ‘Yinzhong’ (Baumeister, 1980; Chen et al., 2004; Zhang et al., 2004, 2005; Kang, 2016), have been bred and widely used in plantations of the Northern Hemisphere owing to their favorable growth performance and pulpwood characteristics (Einspahr, 1984; Zhu et al., 1998; Kang, 2016). In aspect of aneuploidization of *Populus*, interploidy cross is a valid approach. Wang et al. (2017) produced a progeny with extensive segregation of ploidy levels, including many aneuploids, by pollinating triploid ‘Yinzhong’ to diploid *P. tomentosa* × *P. bolleana* clone TB03. For the aneuploids, however, their chromosome compositions were still unclear.

Several methods have been developed to analyze the chromosome compositions of aneuploids, such as cytological karyotypic analysis and marker-based dosage analysis. However, karyotypic analysis based on chromosome morphology is difficult for poplar trees owing to their small and similar chromosomes. By contrast, marker-based dosage analysis has extensive applicability. Esselink et al. (2004) developed a microsatellite DNA allele counting–peak ratios (MAC-PR) method to assign allelic configuration in *Rosa*. This method was also successfully used in the analysis of parental heterozygosity transmission of gametes in *Citrus* (Xie et al., 2015). In *Arabidopsis thaliana*, quantitative fluorescent PCR (QF-PCR) based on microsatellite and InDels markers was used to conduct molecular karyotyping and aneuploidy detection by analyzing the height of highest fluorescent peak or sum of the height of two highest peaks (Henry et al., 2006).

Recently, the fluorescence *in situ* hybridization (FISH) technique was used to position specific chromosomes in meiosis of plants. Zhao et al. (2019) found dramatically biased pairing of homoeologous chromosomes and different univalent and chromosome lagging frequencies between C- and H-subgenomes in interspecific hybrids and allotetraploid *Cucumis* × *hytivus* through oligo-painting and genomic *in situ* hybridization (GISH), concluding that the meiotic behavior harmony of subgenomes is important for meiotic evolution. In *Saccharum officinarum* × *Erianthus arundinaceus* hybrids, unequal chromosome segregation and chromosome losing were observed during meiosis based on 5S rDNA and 45S rDNA FISH analyses (Li et al., 2021). Definitely, the FISH analysis has developed into a reliable tool for tracing the chromosome behavior during meiosis of plants.

In this investigation, aiming to clarify the chromosome composition of each genotype for the TB03 × ‘Yinzhong’ progeny, the MAC-PR method was used to analyze the allelic configurations of each genotype based on the simple sequence repeat (SSR) markers located throughout all 19 chromosomes. The heterozygosity transferred from the male parent was also evaluated based on the allelic configurations for different ploidy groups. Furthermore, cytological records based on 45S rDNA–FISH were conducted to reveal the mechanism of chromosome number variations. These data will help us to understand the rule of chromosome segregation of triploid ‘Yinzhong’.

## Materials and Methods

### Plant Materials

A total of 58 aneuploids were derived from interploidy cross between diploid female parent *Populus tomentosa* × *P. bolleana* clone TB03 and triploid male parent *P. alba* × *P. berolinensis* ‘Yinzhong’ (Wang et al., 2017) were used for chromosome composition analysis in the present study. All plants including parents were cultivated in greenhouse of Beijing Forestry University, Beijing, China. Young leaves were collected from these plants and stored at −80°C for DNA extraction.

### Genomic DNA Extraction and Simple Sequence Repeat Genotyping

Genomic DNA of all samples was isolated from the young leaves using a DP320 DNAsecure Plant Kit (TianGen, Beijing, China) following the manufacturer’s instruction. The quality and concentration of the stock DNA were checked with a NanoDrop 2000 spectrophotometer (Thermo Fisher Scientific, Wilmington, DE, United States). For SSR analysis, all stock DNA samples were diluted to a final concentration of 50 ng μl^–1^ and stored at −20°C.

SSR markers used for this study should be polymorphic between the parents and covers all chromosomes (Xie et al., 2015). According to this principle, 21 markers were selected from a database of the International *Populus* Genome Consortium^1^ and published papers of Yin et al. (2009) and Du et al. (2013). In addition, 26 markers were selected from a self-designed database using the *Populus trichocarpa* genome (v3.1, available at https://phytozome-next.jgi.doe.gov/).

The SSR primer pairs (Supplementary Table 1) were synthesized by Sangon Biotech (Shanghai, China). Following the fluorescence-labeled TP-M13-SSR PCR method (Schuelke, 2000), the forward primer of each pair was tagged with the universal M13 sequence (5′-TGTAAAACGACGGCCAGT-3′) during synthesis. Each PCR used a 20-μl total volume containing 10 μl 2 × TSINGKE^®^ Master Mix (with *Taq*-polymerase Tsingke, Beijing, China), 0.4 pmol forward primer, 1.6 pmol reverse primer, 1.6 pmol fluorescent-dyelabeled (FAM, HEX, TAMRA, ROX) M13 primer (Synthesized by RuiBiotech Inc., Beijing, China), and 15 ng genomic DNA. PCR amplifications were performed in a SimpliAmp™ thermal cycler (Thermo Fisher Scientific, Singapore) using the following program: 94°C for 5 min; followed by 30 cycles of 30 s at 94°C, 45 s at 56°C, and 45 s at 72°C; then 8 cycles of 30 s at 94°C, 45 s at 53°C, and 45 s at 72°C; and a final extension at 72°C for 10 min. Then capillary electrophoresis fluorescence-based SSR analyses were conducted on an ABI 3730xl DNA Analyzer by RuiBiotech Inc., Beijing, China, and the data were analyzed using the GeneMarker software v2.2.0 (SoftGenetics LLC, State College, PA, United States) with the default settings. The relative allelic dosage was estimated based on the MAC-PR method (Esselink et al., 2004) to assess the genotype of each sample.

### 45S rDNA–FISH During Meiosis of ‘Yinzhong’

The male flower buds of ‘Yinzhong’ under meiosis were collected and fixed in Carnoy’s mixture (ethanol:acetic acid = 3:1) for 24 h at 4°C. Subsequently, anthers were harvested to slide preparation. The anthers were washed for 15 min with distilled water and digested using an enzyme mixture, i.e. 3% cellulose Onozuka R-10 (Yakult Pharmaceutical, Tokyo, Japan) and 1% pectolyase Y-23 (Yakult Pharmaceutical, Tokyo, Japan) in 0.01 M citric buffer (pH 4.5), at 37°C for 3 h. Then, the digested anthers were washed for 5 min in distilled water and squashed in a drop of 45% acetic acid on slides. After freezing in liquid nitrogen, the coverslips of the slide preparations were immediately removed. The preparations without coverslips were stored at −20°C for FISH analysis.

According to Li et al. (2001) with some modifications, before *in situ* hybridization, the slide preparations were treated with 100 μg ml^–1^ of DNase-free RNase at 37°C for 1 h and 1 μg ml^–1^ of Proteinase K at 37°C for 40 min. After three 5-min washes in 2 × SSC at room temperature, the slides were post-fixed in 4% paraformaldehyde at room temperature for 10 min, washed twice in 2 × SSC for 5 min, dehydrated through a graded ethanol series (70, 90, and 100%), and air-dried. Plasmids containing 45s rDNA sequence were gifted from Dr. Guixiang Wang in Beijing Academy of Agriculture and Forestry Sciences, China. The 45S rDNA probe was labeled with DIG-11-dUTP using a standard nick translation reaction (DIG-Nick Translation Mix, Roche, Cat. No. 11745816910, Mannheim, Germany).

The fluorescence *in situ* hybridization was performed following standard protocols (Comai et al., 2003). The hybridization mixture contained 50% deionized formamide, 2 × SSC, 10% dextran sulfate, 0.1% SDS, and 2 ng μl^–1^ of labeled probe for each slide. The probe was detected by fluorescein anti-digoxigenin (Anti-Digoxigenin-Fluorescein Fab fragments, Roche, Cat. No. 11207741910, Mannheim, Germany). Chromosomes were counter stained with DAPI in VectaShield antifade mounting medium (Vector Laboratories, Burlingame, CA, United States). All slides were examined using an Olympus BX53 microscope (Olympus, Tokyo, Japan) equipped with a CoolCube 1 camera (MetaSystems, Altlussheim, Germany). Signal capturing and picture processing were performed using the Isis imaging software (MetaSystems, Altlussheim, Germany). The final image adjustment was done with Adobe Photoshop CC 2018 (Adobe Systems Incorporated, San Jose, CA, United States).

### Heterozygosity Analysis

To analyze the genetic heterozygosity of population with mixed ploidy levels, GenoDive v (Meirmans and Van Tienderen, 2004) was used to calculate the observed heterozygosity (*Ho*).

## Results

### Chromosomal Composition in Progeny

**FIGURE 1:**
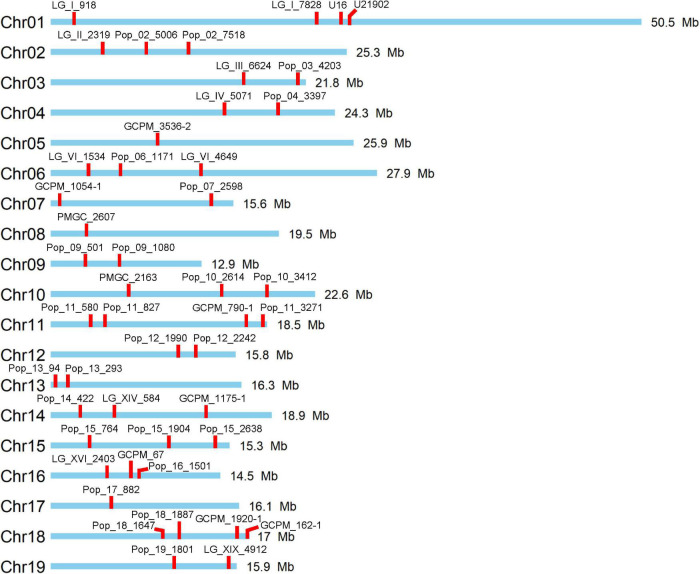
Distribution of used 47 simple sequence repeat (SSR) markers on the 19 chromosomes of *Populus*.

In this study, a total of 47 polymorphic SSR markers with no more than one allele shared between female parent clone TB03 and male parent ‘Yinzhong’ were screened for genotyping of the progeny (Figure 1). Out of the 47 markers, 12 markers dispersed on 11 chromosomes, including LG_I_918, Pop_02_7518, Pop_03_4203, Pop_04_3397, PMGC_2607, PMGC_2163, Pop_11_3271, Pop_13_293, GCPM_67, Pop_18_1647, GCPM_162-1, and Pop_19_1801 amplified completely different fragments between the two parents (Table 1). According to the allele data of the 12 markers in progeny, we found that the female parent TB03 only contributed one locus in all progeny (Supplementary Table 2), suggesting that the female parent TB03 only contributed normal eggs in the interploidy hybridization.

**TABLE 1:** Heterozygosity analysis of each simple sequence repeat (SSR) loci and each chromosome for the mixed ploidy progeny.

Chromosome number	Locus	*Ho* for each allele	*Ho* for each chromosome
Chromosome 1	LG_I_918	0.921	0.830
	LG_I_7828	0.852	
	U16	0.875	
	U21902	0.673	
Chromosome 2	LG_II_2319	0.770	0.718
	Pop_02_5006	0.626	
	Pop_02_7518	0.759	
Chromosome 3	LG_III_6624	0.767	0.858
	Pop_03_4203	0.949	
Chromosome 4	LG_IV_5071	0.905	0.849
	Pop_04_3397	0.793	
Chromosome 5	GCPM_3536-2	0.853	0.853
Chromosome 6	LG_VI_1534	0.876	0.866
	Pop_06_1171	0.832	
	LG_VI_4649	0.891	
Chromosome 7	GCPM_1054-1	0.796	0.722
	Pop_07_2598	0.647	
Chromosome 8	PMGC_2607	0.807	0.807
Chromosome 9	Pop_09_501	0.836	0.830
	Pop_09_1080	0.824	
Chromosome 10	PMGC_2163	0.983	0.869
	Pop_10_2614	0.805	
	Pop_10_3412	0.819	
Chromosome 11	Pop_11_580	0.842	0.891
	Pop_11_827	0.908	
	GCPM_790-1	0.874	
	Pop_11_3271	0.940	
Chromosome 12	Pop_12_1990	0.871	0.855
	Pop_12_2242	0.839	
Chromosome 13	Pop_13_94	0.624	0.796
	Pop_13_293	0.968	
Chromosome 14	Pop_14_422	0.839	0.851
	LG_XIV_584	0.796	
	GCPM_1175-1	0.917	
Chromosome 15	Pop_15_764	0.859	0.782
	Pop_15_1904	0.890	
	Pop_15_2638	0.597	
Chromosome 16	LG_XVI_2403	0.822	0.912
	GCPM_67	0.991	
	Pop_16_1501	0.922	
Chromosome 17	Pop_17_882	0.874	0.874
Chromosome 18	Pop_18_1647	0.989	0.883
	Pop_18_1887	0.830	
	GCPM_1920-1	0.750	
	GCPM_162-1	0.963	
Chromosome 19	Pop_19_1801	0.960	0.934
	LG_XIX_4912	0.908	

**FIGURE 2:**
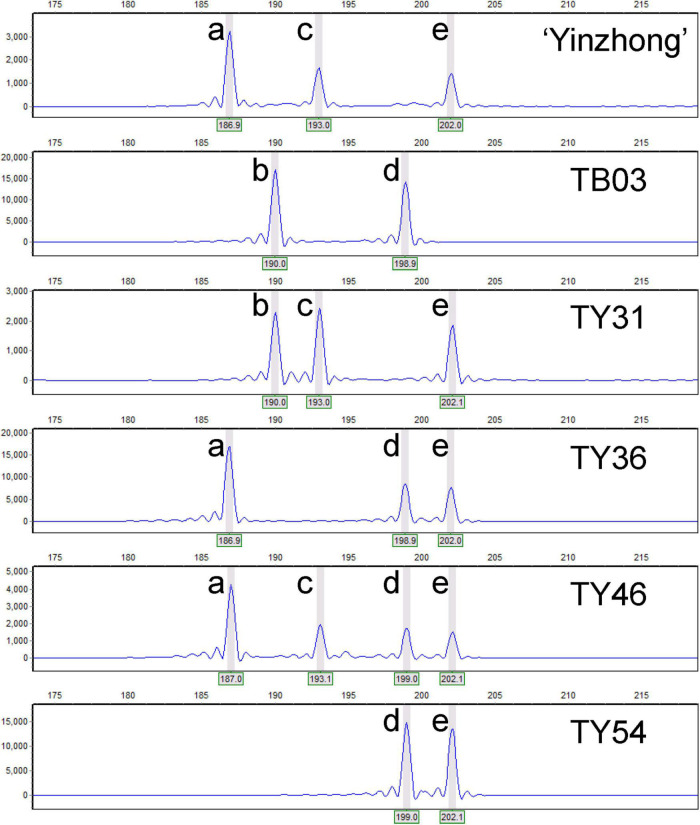
Genotypic analyses of the parents and parts of progeny based on capillary electrophoresis of locus Pop-11-3271. Letters a–e represent the alleles at the locus Pop-11-3271.

**FIGURE 3:**
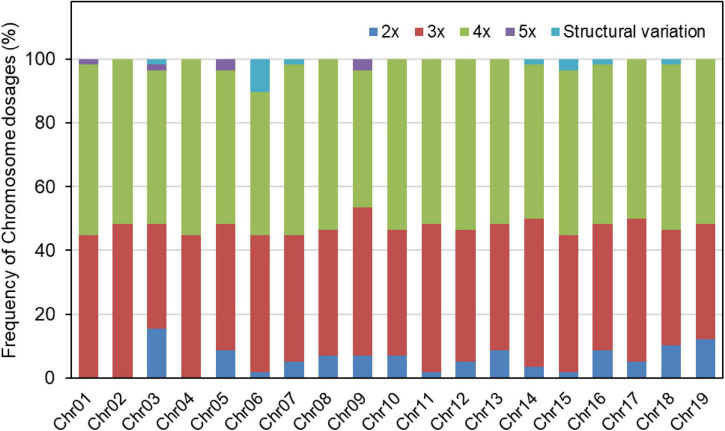
Frequency of each chromosome dosage and occurrence of structural variations in the progeny of TB03 × ‘Yinzhong.’

Based on the relative allelic dosage estimation of MAC-PR analysis at all loci of each sample (Figure 2), chromosomal compositions of all genotypes were revealed. Both chromosomal number variations and structural rearrangements were found in this progeny (Figure 3). In term of chromosomal number variation, all genotypes in the progeny contained at least three copies of chromosomes 1, 2, and 4. The 58 offspring were divided into 26 hypo-triploids, 1 hyper-triploid, 16 hypo-tetraploids, 10 tetraploids, and 5 hyper-tetraploids in the progeny. In the 26 hypo-triploids, the number of missed entire chromosomes ranged from 1 to 5, involved in all chromosomes except chromosomes 1, and 2 to 4. The chromosome 3 was the most frequently missed (∼13.8%) in the hypo-triploids. The unique hyper-triploid contained an extra chromosome 1. In the 16 hypo-tetraploids, the number of missed entire chromosome ranged from 1 to 3, involved in chromosomes 2, 3, 5, 9, 11, 13, 14, 16, 17, and 19. The most frequently missed entire chromosome was chromosome 9, with the frequency of 23.5%. The hyper-tetraploids included 4 4x + 1 type and 1 4x + 2 type. For the 4x + 1 type hyper-tetraploids, the gained chromosomes are involved in chromosomes 3, 5 (in two genotypes), and 9. In the unique 4x + 2 type hyper-tetraploid, there were five copies of chromosomes 1 and 9, respectively.

### Structural Variation Detection

Besides the chromosomal number variation, interestingly, chromosome structural variations, such as duplications and/or deletions, were detected on many loci. For example, in the genotype TY02 (3x-1 type with two chromosome 18), the allelic configure on locus LG_VI_4649 was abc, but the allelic configure on loci LG_VI_1534 and Pop_06_1171 were ac and ab, respectively, suggesting that either deletion occurred on the fragments containing loci LG_VI_1534 and Pop_06_1171 in condition of three copies of chromosome 6 or duplication occurred on the fragment containing locus LG_VI_4649 in condition of two copies chromosome 6. For the genotype TY29 (tetraploid with fragment deletion on chromosome 6), loci on all chromosomes had four copies, except for the chromosome 6. Both the loci LG_VI_1534 and Pop_06_1171 on chromosome 6 showed three alleles and LG_VI_4649 had four alleles, indicating that deletion was happened on chromosome 6. As a result, all tetraploids with deletions of part chromosome were grouped into hypo-tetraploids.

According to the amplified data of total 47 loci, we detected 13 putative structural variation events in 12 genotypes. The genotype TY02 included 2 structural variation events, locating on chromosomes 6 and 7, respectively. The 13 structural variation events were dispersed on 7 different chromosomes, i.e., chromosomes 3, 6, 7, 14, 15, 16, and 18 (Figure 3). Six structural variation events occurred on chromosome 6, accounting for 46.2%, and two events occurred on chromosome 15.

### Analysis of Heterozygosity Transmission

According to allelic variation of the 47 loci, the observed heterozygosity of the whole progeny was 0.843. The heterozygosity levels of loci were positively related with the number of alleles (*r* = 0.8264, *p* = 8.494e-13). For the different chromosomes, the maximum *Ho* was 0.934 on chromosome 19 and the minimum was 0.718 on chromosome 2 (Table 1). The *Ho* of the hypo-triploids, hyper-triploid, hypo-tetraploids, tetraploids, and hyper-tetraploids was 0.844, 0.862, 0.846, 0.835, and 0.843, respectively.

In the aspect of paternal heterozygosity transmission, the transmitted paternal heterozygosity in the progeny was 0.795. Based on the chromosome-specific analysis, the maximum paternal heterozygosity transmission was 0.925 of chromosome 17 and the minimum was 0.477 of chromosome 8 (Supplementary Table 3). The locus with the highest paternal heterozygosity transmission was LG_IV_5071 on chromosome 4 and the locus with the lowest paternal heterozygosity transmission was Pop_02-7518 on chromosome 2. The transmitted paternal heterozygosity in the hypo-triploids, hyper-triploid, hypo-tetraploids, tetraploids, and hyper-tetraploids was 0.748, 0.887, 0.830, 0.833, and 0.836, respectively, indicating that the viable pollen gains of ‘Yinzhong’ were able to transmit high heterozygosity to progeny.

### Meiotic Specific-Chromosome Segregation Feature

**FIGURE 4:**
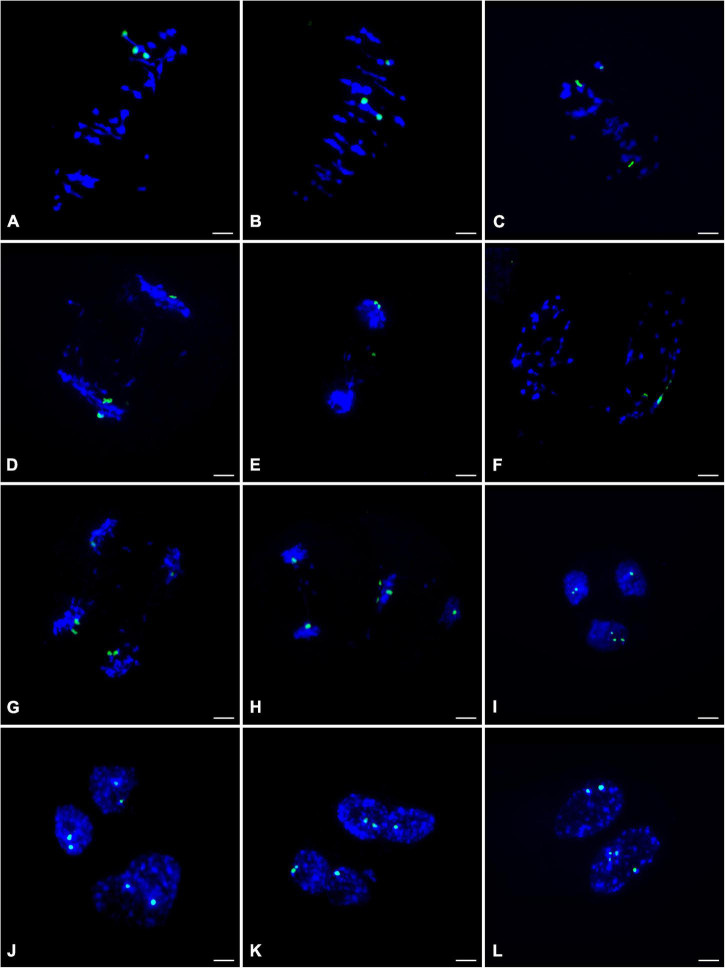
45S rDNA– fluorescence *in situ* hybridization (FISH) analysis during the meiosis of the male parent ‘Yinzhong’. **(A)** Metaphase I cell showing one trivalent with 45S rDNA signals (green). **(B)** Metaphase I cell showing a bivalent and a univalent with 45S rDNA signals. **(C)** Metaphase I cell showing three univalents with 45S rDNA signals. **(D)** Anaphase I cell showing imbalance 45S rDNA-located chromosome segregation. **(E)** Anaphase I cell showing one 45S rDNA signal on a lagging chromosome. **(F)** Telophase I cell showing all 45S rDNA signals in one daughter nucleus, **(G)** Anaphase II cell with normal meiotic 45S rDNA-located sister chromatid segregation. **(H)** Anaphase II cell showing three 45S rDNA signals in one chromosome group. **(I)** Two normal daughter nuclei at telophase II including one and two 45S rDNA signals, respectively, and one fused nucleus including three signals. **(J)** Two normal daughter nuclei and one fused nucleus at telophase II including two 45S rDNA signals, respectively. **(K)** Telophase II with two fused nuclei including three 45S rDNA signals, respectively. **(L)** Telophase II with two fused nuclei including two and four 45S rDNA signals, respectively.

To clarify the chromosome segregation feature during meiosis of ‘Yinzhong,’ chromosome behaviors were observed based on 45S rDNA–FISH analysis. At metaphase I of ‘Yinzhong,’ the pairing pattern of the 45S rDNA located chromosomes exhibited univalent (I) + bivalent (II), I + I + I, and trivalent (III) types (Figures 4A–C), suggesting that the 45S rDNA signals were just located three homoeologous chromosomes of ‘Yinzhong’. At anaphase I, the segregation of the chromosomes commonly followed the unequal 1:2 pattern in most of the cells (Figure 4D). One of the 45S rDNA signals also might locate on a lagging chromosome in some cells (Figure 4E). Interestingly, several cells with three 45S rDNA signals located in one daughter nucleus were observed (Figure 4F).

At anaphase II, the sister chromatids were usually segregated into daughter nuclei (Figure 4G). However, failed segregation of sister chromatids also was observed in several microcytes, resulting in two sister chromatids moved into the same daughter nucleus (Figure 4H). At telophase II, although microcytes with normal four daughter nuclei were dominant, microcytes with fused nuclei were also observed. In normal daughter nucleus, the number of 45S rDNA signals was 0–3, and in the fused nucleus, 2–4 45S rDNA signals were recorded (Figures 4I–L), suggesting that 0–4 dosages of 45S rDNA located chromosome will be contained in the male gametes of ‘Yinzhong.’

## Discussion

Microsatellite DNA allele counting–peak ratio has been proved as a feasible method for the allelic configuration analysis of polyploids in *Rosa* and *Citrus* (Esselink et al., 2004; Babaei et al., 2007; Cuenca et al., 2011; Xie et al., 2015). In the present study, the MAC-PR method was successfully used in quantification of the allele copy number of *Populus* aneuploids. According to the allele copy number of each SSR locus, the genotypes of all offspring were determined. Furthermore, the chromosomal composition and occurrence of structural variation for each genotype were clarified. Compared to cytological karyotyping based on the chromosome number and morphology, the molecular karyotyping based on the MAC-PR method exhibited higher efficiency and easier operation.

Interploidy cross is an important pathway for the creation of new germplasms with ploidy variations, although it is usually accompanied by highly reproductive barrier (Birchler, 2014; Alexander, 2020). In *Actinidia chinensis*, triploid, tetraploid, pentaploid, heptaploid, and octaploid hybrids were produced by interploidy cross between hexaploid and diploid parents (Rao et al., 2012). In the previous study on *Populus*, Wang et al. (2017) produced a progeny with segregated ploidy levels, including many aneuploids, by pollinating triploid ‘Yinzhong’ to diploid TB03, because of the imbalanced meiotic chromosomal segregation of the ‘Yinzhong’. In the present study, the chromosomal compositions of each genotype in the TB03 × ‘Yinzhong’ progeny were further revealed by the MAC-PR analysis. Forty-eight of 58 genotypes were aneuploids with various chromosomal compositions, including 26 hypo-triploids, 1 hyper-triploid, 16 hypo-tetraploids, and 5 hyper-tetraploids. No hypo-diploid was found, suggesting that gamete without complete chromosome set might be sterile in *Populus* or rare hypo-diploid was aborted.

In general, triploids undergo normal meiosis to give rise to aneuploid gametes with approximate 3x/2 chromosome number. However, because of the imbalanced chromosome number, the aneuploid gametes are almost sterile (Ramsey and Schemske, 1998; Charles et al., 2010). In the present study, 26 hypo-triploids (3x–1∼5) and 1 hyper-triploid (3x + 1) were determined from the progeny of diploid “TB01” × triploid ‘Yinzhong,’ which probably attributed to fertilization of rarely fertile aneuploid pollen grains from normal meiosis of the male parent ‘Yinzhong’. All aneuploids had chromosome numbers more than x + 3x/2, probably suggesting that the aneuploid pollen grains with chromosome number less than 3x/2 might had lower viability than that with more than 3x/2 chromosomes. The chromosome 3 was the most frequently missed (∼34.6%) in the hypo-triploids, which might suggest that the development of aneuploid embryo is not very sensitive to the copies of chromosome 3.

In a previous study, the production of unreduced pollen grains was observed in ‘Yinzhong’ (Wang et al., 2017). The formation of tetraploids in our study could be explained by union between normal x egg and unreduced 3x sperm. Because the imbalanced chromosome segregation commonly happens at the first meiotic division of triploids, theoretically, triploid can produce fertile unreduced 3x gametes through first meiotic division restitution (FDR) but not second meiotic division restitution (SDR). Therefore, we speculated that the unreduced pollen grains from triploid ‘Yinzhong’ should be FDR type. Additionally, chromosomes elimination was also observed during the meiosis of ‘Yinzhong’ (Wang et al., 2017), which could produce aneuploid unreduced pollen grains with loss of several chromosomes when FDR took place. In this study, the production of the hypo-tetraploids could attribute to fertilization of the aneuploid unreduced pollen grains.

It is interesting that 5 hyper-tetraploids with 1–2 extra chromosomes were determined in the progeny, suggesting that viable male gametes containing 3 chromosome sets plus 1–2 extra chromosomes took part in fertilization. The FISH analysis showed that three 45S rDNA signals were found on three homoeologous chromosomes at metaphase I stage of male parent ‘Yinzhong’ microsporocytes and 2–4 45S rDNA signals could be observed in fused daughter nucleus of telophase II. The fused nucleus with 4 signals probably contained unreduced chromosome number and extra one 45S rDNA signals located chromosome. Therefore, the formation of the male gametes containing unreduced chromosome number and extra chromosomes could be attributed to imbalanced meiotic chromosomal segregation and division restitution of the male parent triploid ‘Yinzhong’. According to Xin et al. (2020), the 45S rDNA FISH signals were positioned on chromosomes 8 and 14 for *P. lasiocarpa*, *P. deltoides*, and *P. trichocarpa*, on chromosome 14 for *P. tremula* × *P. tremuloides*, and *P. tomentosa*, and on chromosome 9 for *P. euphratica*. The ‘Yinzhong’ is an intersectional hybrid between sect. Populus and sect. Aigeiros (Zhou et al., 1992); so, we speculated that the chromosomes with the 45S rDNA signals were chromosome 8 or chromosome 14. Although the 45S rDNA located chromosome was not corresponding to the extra chromosome in the hyper-tetraploids in the present study, the FISH analysis still gave us a reasonable explanation for the cytological mechanism of the hyper-tetraploid formation.

Chromosome structural variation is an important type of chromosome aberrations, which can drive species evolution and promote plant improvement (Wijnker and de Jong, 2008; Yang et al., 2012; Danilova et al., 2017). Abnormal meiosis of allopolyploids usually results in the production of chromosome structural variations (Gou et al., 2018). In our study, chromosome structural variations, such as duplications and deletions, were detected by SSR molecular markers on many loci. Occurrence of chromosomal rearrangements is related with chromosome breakpoint regions (Lysák and Schubert, 2013). The present study showed that 46.2% structural variation events occurred on chromosome 6, probably suggesting that a chromosome breakpoint locates near the loci of chromosome 6. However, it is difficult to detect the occurrence of all structural variations, owing to the limited number and density of SSR markers. The inversion and translocation structural variation types are also impossible to be determined by SSR marker method. Molecular cytological tools, such as GISH and FISH, are effective methods to detect occurrence of chromosome structural rearrangements in plants (Lou et al., 2014; Danilova et al., 2017; Huang et al., 2018; Said et al., 2019, 2021). Lou et al. (2014) developed single-copy gene pools in *Cucumis sativus*, and analyzed chromosome rearrangements in the *Cucumis* genus by using single-copy gene-based chromosome painting method. In addition, molecular karyotyping technique depending on read-counting of DNA sequencing also was used to precisely detect abundant deletions and insertions of chromosomal segments in poplar progeny derived from crossing with gamma-irradiated pollen (Henry et al., 2015). Therefore, in order to completely analyze the structural variations in our materials, the chromosome painting and read-counting-based molecular karyotyping methods could be applied.

According to the mechanism of unreduced gamete formation, the FDR type unreduced gametes contain non-sister chromatids, which can transmit approximately 80% parental heterozygosity theoretically when crossovers occur; the SDR type unreduced gametes contain sister chromatids, which can transmit approximately 40% parental heterozygosity (Hermsen, 1984). In induced 2n eggs of *Populus*, it was found that the FDR, SDR, and postmeiotic restitution types 2n eggs transmitted 0.7480, 0.3958, and 0.3590 maternal heterozygosities, respectively (Dong et al., 2015). As the above inference, the tetraploids, hypo-tetraploids, and hyper-tetraploids should be derived from the hybridization of euploid or aneuploid unreduced pollen grains in this study. Their transmitted paternal heterozygosities reached 0.830, 0.833, and 0.836, which support our conclusion of the unreduced pollen of male parent ‘Yinzhong’ were FDR type.

Numerical and structural variations in chromosomes commonly result in complex phenotypic trait changes. In *Arabidopsis* aneuploids, phenotypic variation is strongly associated with chromosomal composition and dosage (Henry et al., 2010). Bastiaanse et al. (2019, 2021) found that gene dosage variations in *Populus* carrying large DNA fragmental insertions and deletions can influence morphological variation through complex changes in gene expression, and identified some dosage-sensitive genomic regions that influenced pleiotropic morphological characters. The vessel morphology in *Populus* was also affected by gene dosages (Rodriguez-Zaccaro et al., 2021). In our study, chromosomal composition and structural variation of each aneuploid for the full-sib progeny of TB03 × ‘Yinzhong’ were clarified, which provides a potential material for analyzing mechanisms of trait variation relying on chromosome or gene dosages. In future, an exact analysis of gene dosage variations in the aneuploids could be conducted by the read-counting-based molecular karyotyping method. Effects of gene dosage variations on trait changes of *Populus* also could be deciphered by association analysis between gene copy numbers and trait phenotypes. Additionally, after flowering, we hope that the aneuploids can play a valuable role in chromosome engineering breeding of *Populus*.

## Data Availability Statement

The datasets presented in this study can be found in online repositories. The names of the repository/repositories and accession number(s) can be found in the article/Supplementary Material.

## Author Contributions

JW conceived and designed the research. Y-HZ, Y-FZ, and Y-XX conducted the SSR experiments and analyzed the data. L-JW conducted FISH experiments. J-WZ cultivated the materials. Y-HZ and JW wrote the manuscript. All authors read and approved the manuscript.

## Conflict of Interest

The authors declare that the research was conducted in the absence of any commercial or financial relationships that could be construed as a potential conflict of interest.

## Publisher’s Note

All claims expressed in this article are solely those of the authors and do not necessarily represent those of their affiliated organizations, or those of the publisher, the editors and the reviewers. Any product that may be evaluated in this article, or claim that may be made by its manufacturer, is not guaranteed or endorsed by the publisher.

## Footnotes

1
http://web.ornl.gov/sci/ipgc/ssr_resource.htm
