# Supplementary material for: Variation of Chromosome Composition in a Full-Sib Population Derived From 2x × 3x Interploidy Cross of Populus
Source: Front Plant Sci. 2022 Jan 26;12:816946. doi: 10.3389/fpls.2021.816946 (PMC8825477; doi:10.3389/fpls.2021.816946)
Supplement: Supplementary file 1 [file Table_1.docx]

**Supplementary Table 1** Information of SSR markers used in this study.

| Locus | Location | Motif | Position | Primer sequences (5’🡪 3’) | Genotype of ‘YZY’ | Genotype of ‘TB03’ |
| --- | --- | --- | --- | --- | --- | --- |
| LG_I_918 | Chr01 | (GAA)12 | 1986206 | F: GGCAACTCAGATTGATGATT  R: TCTTGTTCGATCTGGAGAGT | abc | de |
| LG_I_7828 | Chr01 | (GAG)14 | 22628636 | F: TCCTCTTCTAAACAACCCAA  R: TTCTCGTTTAGAATCTTCGC | bcd | ac’ |
| U16 | Chr01 | (GAG)9 | 24596426 | F: GGAGGACCAGATAAGGGAGC  R: TGGGGTAAGCTGACTTGCTT | acd | a’b |
| U21902 | Chr01 | (TCC)7 | 25475955 | F: CAGTCCTCAAGGCTGAATCC  R: ACTCGATGTCAACCGAGCTT | abb | b’c |
| LG_II_2319 | Chr02 | (GTT)9 | 4442567 | F: TGATTGTAGAGAAGCCACCT  R: TGCATTGTTTCAGTCTCAAG | abc | a’a’ |
| Pop_02_5006 | Chr02 | (CTT)6 | 8134101 | F: CTGTTGTTCATGGGGTTTGA  R: TCCGGATAAACCAAAAGGAA | abb | a’c |
| Pop_02_7518 | Chr02 | (AC)9 | 11950337 | F: TAAGAATGCCCAACCCATCT  R: TTGTCAGGTACCGATGATGC | bbd | ac |
| LG_III_6624 | Chr03 | (GAA)6 | 16426318 | F: ATGAAGATGAAAGAGTGCGT  R: CTGTTTCCATCCTTTGACAT | abc | a’a’ |
| Pop_03_4203 | Chr03 | (GGA)9 | 21769145 | F: AGTTGGTGGTGGAGGAAGTG  R: AGAAGGGAACGATGATGGTG | abc | de |
| LG_IV_5071 | Chr04 | (GA)11 | 14764305 | F: CCTTTCTGTATTGTCCTTCG  R: TTGAATACCAAAGGCATCTT | acd | bd’ |
| Pop_04_3397 | Chr04 | (AAG)6 | 19513205 | F: AAGCAGCTAAAATGGACGGA  R: CCTCCCTTGTGGCTAAATCA | bbc | aa |
| GCPM_3536-2 | Chr05 | (TTG)7 | 9138746 | F: AGATTCTTTTTCGGCTTCTT  R: AGAAGATGCTGGAGTTCAGA | abd | cd’ |
| LG_VI_1534 | Chr06 | (GGA)6 | 3345048 | F: GTAAACTCCGGTTCATGGTA  R: GTATAACAACAGCGAGGGAG | abc | c’d |
| Pop_06_1171 | Chr06 | (GAT)6 | 6046358 | F: CCTTGCACGCTGATGAAGTA  R: CCATTGACATGATGGACCAA | acd | bd’ |
| LG_VI_4649 | Chr06 | (TTG)8 | 12937836 | F: GGTGTAGTTAAGCGATTTGG  R: ACTGAACCCTCTTCTTCTCC | acd | a’b |
| GCPM_1054-1 | Chr07 | (CT)11 | 863475 | F: AGGTCTGTGCAAGGAATAAA  R: GTCTGTAATCAAGCCAAAGC | abd | b’c |
| Pop_07_2598 | Chr07 | (TCG)7 | 13919399 | F: GCCAGAAAGAGAAGGTGACG  R: CGATGGCGAAGAAGAAACTC | bcc | ac’ |
| PMGC_2607 | Chr08 | GA | 3071838 | F: TTAAAGGGTGGTCTGCAAGC  R: CTTCTTGCACCTCGTTTTGAG | acc | bd |
| Pop_09_501 | Chr09 | (GAA)6 | 3006866 | F: CCCAGAAGAGGATTAAGGGC  R: CCAGCAGCAATGGTTGAGTA | abd | a’c |
| Pop_09_1080 | Chr09 | (CAC)5 | 5962485 | F: GAGGAAGAGTAATCGCGCAG  R: TTTAATTCTGCCTTGGTGCC | abc | c’d |
| PMGC_2163 | Chr10 | GA | 6657656 | F: CAATCGAAGGTAAGGTTAGTG  R: CGTTGGACATAGATCACACG | acd | bb |
| Pop_10_2614 | Chr10 | (ATA)6 | 14549438 | F: GTAAGCGGTATTAGCAGCGG  R: GAAACTGTGGAGGACGGAGA | abc | c’d |
| Pop_10_3412 | Chr10 | (AG)13 | 18525456 | F: GATGGGCCAACAAGATGATT  R: CTCGGACGAGGAAATAGTGG | abd | b’c |
| Pop_11_580 | Chr11 | (TTC)5 | 3425742 | F: GAGGGACATGGAGTGCTGAT  R: TTCCCTGACCTTGACAAACC | acd | a’b |
| Pop_11_827 | Chr11 | (AAC)13 | 4669113 | F: AGCCCGGTGACTCCTTTAAT  R: GGGCTCAGTCTCTCTTGGTG | abd | b’c |
| GCPM_790-1 | Chr11 | (ATA)6 | 16773169 | F: GACCAAAACTTCACTGCTTC  R: CCAATTGAAGTTCACGGTAT | bcd | ab’ |
| Pop_11_3271 | Chr11 | (CAT)9 | 18449001 | F: TCCAGGAGGGCAGATTTATG  R: GTGAATTGTGATCGATTTGACC | ace | bd |
| Pop_12_1990 | Chr12 | (TTC)5 | 11016908 | F: ATGGGATTCTTTGAGCGATG  R: CCTTTCCATTTGGGCAATAA | acd | bc’ |
| Pop_12_2242 | Chr12 | (AAG)7 | 12368862 | F: TTTGGGGCCACAATCTAAAG  R: GCTGCTGATCGCTCTCTCTT | acd | bd’ |
| Pop_13_94 | Chr13 | (AGA)8 | 414068 | F: TATTGGAATTGGATCCACGC  R: GGTTAATCGAGCCCCAAAAT | abb | b’c |
| Pop_13_293 | Chr13 | (CAT)6 | 1667798 | F: TGTTGGCAAAACTGAGACCA  R: CAAGCCTAGAATCCCAAGACA | bce | ad |
| Pop_14_422 | Chr14 | (GAG)5 | 2504462 | F: ACCCTTCAACAACAACCCAA  R: CAAAACCCACCAAGCAACTT | bcd | ab’ |
| LG_XIV_584 | Chr14 | (AT)13 | 5527165 | F: TACTGGTGGTGCTCAATACA  R: AAAGCAAACGCAGTAATAGC | bcd | ab’ |
| GCPM_1175-1 | Chr14 | (CTT)11 | 13328847 | F: TCATCAACCTGACCTCTACC  R: CAAAGCAAAAACAAACACAA | acd | bc’ |
| Pop_15_764 | Chr15 | (TC)10 | 3432307 | F: TGCACATCAACACCAAGAAA  R: CCATCTCACCCTCTCCCTCT | abc | a’d |
| Pop_15_1904 | Chr15 | (TG)8 | 10166131 | F: CACACCCAAGTCACATCGAA  R: CAGGTAGTTGGCTAATAGAGCTTTG | abd | cd’ |
| Pop_15_2638 | Chr15 | (AC)7 | 14140358 | F: CACCATTGGATTCCCTCAAA  R: TTTTGGGTTACGTGCTGTTG | abb | b’c |
| LG_XVI_2403 | Chr16 | (CT)9 | 4981910 | F: CTTCTAAACAGCTGGTGGAC  R: CTCAGACCCAGAATGACACT | abd | cd’ |
| GCPM_67 | Chr16 | (AAT)10 | 6967825 | F: TGAAGCCCTCACTACTCATT  R: CCCCAATCTTTTGTTTATTG | ace | bd |
| Pop_16_1501 | Chr16 | (TGC)6 | 7602527 | F: GTTCGTCTCGCAGCTCTTCT  R: TGGAGCAAAACCAGGGTTAC | abd | a’c |
| Pop_17_882 | Chr17 | (AAG)6 | 5251183 | F: AGGACATGCATAGACACACATT  R: CAGAATGCAATCGTCTTGTGA | abd | b’c |
| Pop_18_1647 | Chr18 | (AG)6 | 9737588 | F: GCCAGAGCCTCACAAATCAT  R: TGATGCATGAAAACAGTGGC | bde | ac |
| Pop_18_1887 | Chr18 | (GAA)5 | 11146891 | F: TAGCATTGGTGACTGGGACA  R: ACCAATGCCTTAATTCGTCG | acd | bd’ |
| GCPM_1920-1 | Chr18 | (CAA)6 | 16036678 | F: AGTTTGAATCATGCTGGTCT  R: TTTACACTCATTGAACCGAA | abc | b’b’ |
| GCPM_162-1 | Chr18 | (CTT)6 | 16875460 | F: GCCCAAACTCTTATTTGATG  R: TGGTGGAGGCTAGGATAGTA | abd | cc |
| Pop_19_1801 | Chr19 | (AG)10 | 10563824 | F: AAAAGGATCAGGCAAAACGA  R: ATGGGTATGGCGTCATGATT | abc | de |
| LG_XIX_4912 | Chr19 | (CA)11 | 15341539 | F: GTCGTTGTTAGGGTTAAACG  R: ATATGCCACATATACGCACA | acd | a’b |
