# Supplementary material for: Variation of Chromosome Composition in a Full-Sib Population Derived From 2x × 3x Interploidy Cross of Populus
Source: Front Plant Sci. 2022 Jan 26;12:816946. doi: 10.3389/fpls.2021.816946 (PMC8825477; doi:10.3389/fpls.2021.816946)
Supplement: Supplementary file 3 [file Table_3.DOCX]

**Supplementary Table 3 Paternal heterozygosity transmission by each SSR locus and each chromosome in the mixed ploidy progeny.**

| Chromosome | Locus | *Ho* for each allele | *Ho* for each chromosome |
| --- | --- | --- | --- |
| Chromosome 01 | LG_I_918 | 0.813 | 0.821 |
|  | LG_I_7828 | 0.894 |  |
|  | U16 | 0.911 |  |
|  | U21902 | 0.664 |  |
| Chromosome 02 | LG_II_2319 | 0.851 | 0.542 |
|  | Pop_02_5006 | 0.391 |  |
|  | Pop_02_7518 | 0.385 |  |
| Chromosome 03 | LG_III_6624 | 0.744 | 0.739 |
|  | Pop_03_4203 | 0.733 |  |
| Chromosome 04 | LG_IV_5071 | 0.989 | 0.748 |
|  | Pop_04_3397 | 0.506 |  |
| Chromosome 05 | GCPM_3536-2 | 0.825 | 0.825 |
| Chromosome 06 | LG_VI_1534 | 0.882 | 0.875 |
|  | Pop_06_1171 | 0.819 |  |
|  | LG_VI_4649 | 0.925 |  |
| Chromosome 07 | GCPM_1054-1 | 0.799 | 0.716 |
|  | Pop_07_2598 | 0.632 |  |
| Chromosome 08 | PMGC_2607 | 0.477 | 0.477 |
| Chromosome 09 | Pop_09_501 | 0.848 | 0.844 |
|  | Pop_09_1080 | 0.839 |  |
| Chromosome 10 | PMGC_2163 | 0.885 | 0.812 |
|  | Pop_10_2614 | 0.828 |  |
|  | Pop_10_3412 | 0.724 |  |
| Chromosome 11 | Pop_11_580 | 0.928 | 0.897 |
|  | Pop_11_827 | 0.902 |  |
|  | GCPM_790-1 | 0.908 |  |
|  | Pop_11_3271 | 0.851 |  |
| Chromosome 12 | Pop_12_1990 | 0.868 | 0.857 |
|  | Pop_12_2242 | 0.845 |  |
| Chromosome 13 | Pop_13_94 | 0.471 | 0.655 |
|  | Pop_13_293 | 0.839 |  |
| Chromosome 14 | Pop_14_422 | 0.839 | 0.868 |
|  | LG_XIV_584 | 0.833 |  |
|  | GCPM_1175-1 | 0.931 |  |
| Chromosome 15 | Pop_15_764 | 0.920 | 0.764 |
|  | Pop_15_1904 | 0.940 |  |
|  | Pop_15_2638 | 0.431 |  |
| Chromosome 16 | LG_XVI_2403 | 0.851 | 0.881 |
|  | GCPM_67 | 0.879 |  |
|  | Pop_16_1501 | 0.914 |  |
| Chromosome 17 | Pop_17_882 | 0.925 | 0.925 |
| Chromosome 18 | Pop_18_1647 | 0.874 | 0.839 |
|  | Pop_18_1887 | 0.839 |  |
|  | GCPM_1920-1 | 0.822 |  |
|  | GCPM_162-1 | 0.822 |  |
| Chromosome 19 | Pop_19_1801 | 0.793 | 0.793 |
|  | LG_XIX_4912 | 0.793 |  |
